# Differential Recognition of Clinically Relevant *Sporothrix* Species by Human Granulocytes

**DOI:** 10.3390/jof9100986

**Published:** 2023-10-04

**Authors:** Ana K. Galván-Hernández, Manuela Gómez-Gaviria, Iván Martínez-Duncker, José A. Martínez-Álvarez, Héctor M. Mora-Montes

**Affiliations:** 1 Departamento de Biología, División de Ciencias Naturales y Exactas, Campus Guanajuato, Universidad de Guanajuato, Noria Alta s/n, col. Noria Alta, C.P., Guanajuato Gto. 36050, Mexico; ak.galvanhernandez@ugto.mx (A.K.G.-H.); m.gomezgaviria@ugto.mx (M.G.-G.); martinezjose@ugto.mx (J.A.M.-Á.); 2 Laboratorio de Glicobiología Humana y Diagnóstico Molecular, Centro de Investigación en Dinámica Celular, Instituto de Investigación en Ciencias Básicas y Aplicadas, Universidad Autónoma del Estado de Morelos, Cuernavaca Mor. 62209, Mexico; duncker@uaem.mx

**Keywords:** fungal cell wall, cytokine production, phagocytosis, neutrophil extracellular traps, *N*-linked glycans, *O*-linked glycans, β-1,3-glucan, innate immune sensing

## Abstract

Sporotrichosis is a cutaneous mycosis that affects humans and animals and has a worldwide distribution. This infection is mainly caused by *Sporothrix schenckii*, *Sporothrix brasiliensis*, and *Sporothrix globosa*. Current research about anti-*Sporothrix* immunity has been mainly focused on *S. schenckii* and *S. brasiliensis*, using different types of human or animal immune cells. Granulocytes are a group of cells relevant for cytokine production, with the capacity for phagocytosis and the generation of neutrophil extracellular traps (NETs). Considering their importance, this study aimed to compare the capacity of human granulocytes to stimulate cytokines, uptake, and form NETs when interacting with different *Sporothrix* species. We found that conidia, germlings, and yeast-like cells from *S. schenckii*, *S. brasiliensis*, and *S. globosa* play an important role in the interaction with these immune cells, establishing morphology- and species-specific cytokine profiles. *S. brasil-iensis* tended to stimulate an anti-inflammatory cytokine profile, whilst the other two species had a proinflammatory one. *S. globosa* cells were the most phagocytosed cells, which occurred through a dectin-1-dependent mechanism, while the uptake of *S. brasiliensis* mainly occurred via TLR4 and CR3. Cell wall *N*-linked and *O*-linked glycans, along with β-1,3-glucan, played a significant role in the interaction of these *Sporothrix* species with human granulocytes. Finally, this study indicates that conidia and yeast-like cells are capable of inducing NETs, with the latter being a better stimulant. To the best of our knowledge, this is the first study that reports the cytokine profiles produced by human granulocytes interacting with *Sporothrix* cells.

## 1. Introduction

Sporotrichosis is a cutaneous and subcutaneous mycosis caused by members of the *Sporothrix* genus, which contain pathogenic and environmental species [1,2]. The etiological agents are mostly prevalent in tropical and subtropical areas, with epidemic areas reported in Mexico, Peru, Brazil, South Africa, India, and China, among others [3,4]. Different from other mycoses, sporotrichosis is not specific to human beings and can affect wild and domestic mammals, such as cats and dogs, which are sources of fungal agents; therefore, the disease is considered a zoonosis [5,6,7]. Most sporotrichosis cases are benign lymphocutaneous infections that do not compromise the patient’s life; however, the disseminated form that affects deep-seated organs is likely to occur in immunocompromised patients and is associated with high mortality rates [3,5]. Fixed cutaneous infection is another frequent form of the disease, and in this case, the infection is auto-limited most likely because of an immune response that avoids the dissemination of the pathogen to other organs [8].

The most frequently isolated species from sporotrichosis cases are *Sporothrix schenckii*, *Sporothrix brasiliensis*, and *Sporothrix globosa* [9]. *S. brasiliensis* has recently stood out from the other two species because of the alarming epidemic outbreak of animal and human sporotrichosis that originally started in Brazil but recently expanded to other South American countries; in contrast, *S. globosa* is mostly isolated in China and other Asian countries [10]. The three species are thermodimorphic and grow in the environment as mycelia, which produce conidia. These fungal morphologies are the ones that infect host tissues and, once adapted to body temperature, undergo dimorphism to yeast-like cells, a morphology associated with dissemination to tissues and organs [7]. However, this classic division of morphology and stage of the infective cycle have been challenged by recent observations: germlings and hyphae have been observed in human and animal cases of sporotrichosis [11,12,13], and yeast-like cells can be transmitted from infected animals to healthy animals and human beings [14,15].

The study of anti-*Sporothrix* immunity has attracted attention in recent years in an attempt to understand the basis of the differences displayed by these species when interacting with host tissues and because it is an essential component in the search for immunomodulatory approaches helping in the treatment of sporotrichosis. Moreover, sporotrichosis is one of the few mycoses where an antibody-based immunity is capable of protecting the host from the infection [16,17]. Thus far, both adaptive and innate immunity against *Sporothrix* have been studied, but the latter has been studied to a greater extent and mainly in terms of when immune effectors interact with *S. schenckii* or *S. brasiliensis* [18,19]. Thus far, the interaction of complement, peripheral blood mononuclear cells (PBMCs), macrophages, dendritic cells, and NK cells with *Sporothrix* cells has been reported [20,21,22,23,24,25], but there is limited information about the contribution of granulocytes to anti-*Sporothrix* immunity. This group of cells is a relevant cytokine producer and can phagocyte and generate extracellular traps, actions that contribute to one of the first attempts to control pathogens by innate immunity cells [26].

It is known that human polymorphonuclear leukocytes can phagocyte *S. schenckii* yeast-like cells in the presence of complement [27], and this observation was further supported by histological analyses of human sporotrichosis cases [28]. In a comparative study, *S. schenckii* yeast-like cells were more phagocytosed than conidia, but fungal viability was not significantly affected [29]. Moreover, soluble extracellular components of *S. schenckii* cultures were capable of stimulating more reactive oxygen species in human granulocytes than *Candida albicans* preparations, suggesting a more potent proinflammatory response against *S. schenckii* [30].

Here, we compared the ability of human granulocytes to stimulate cytokines, to uptake, and to form neutrophil extracellular traps (NETs) when interacting with conidia, yeast-like cells, or germlings from *S. schenckii*, *S. brasiliensis*, or *S. globosa*. Moreover, we also analyzed the contribution of some pattern recognition receptors and cell wall components during the interaction of these fungal cells with human granulocytes.

## 2. Materials and Methods

### 2.1. Strains and Culturing Conditions

Strains ATCC MYA-4821, ATCC MYA 4823, and FMR 9624 from *S. schenckii*, *S. brasiliensis*, and *S. globosa*, respectively, were used in this work. The three strains are clinical isolates previously characterized at the species level via molecular techniques and are reference strains for both genetic and phenotypic analyses [31,32,33,34,35]. Mycelia was grown in YPD broth, pH 4.5 (1% [*w*/*v*] yeast extract, 2% [*w*/*v*] gelatin peptone, and 3% [*w*/*v*] dextrose), at 28 °C. For solid plates, 2% [*w*/*v*] agar was included in the medium composition. After seven days of incubation on a solid medium, 10 mL of deionized water was added to detach conidia, and these were collected via aspiration and used for the induction of other morphologies or in interactions with human cells [36]. To obtain germlings, conidia were incubated for 11–12 h in YPD, pH 4.5, at 28 °C and underwent shaking at 120 rpm, while dimorphism to yeast-like cells was induced by placing conidia in YPD, pH 7.8, and incubating them for four days at 37 °C and 120 rpm [20]. All morphotypes were washed six times with chilled PBS and immediately used for cell wall modifications or interactions with human cells. To assess the contribution of cell wall glycans to the interactions with human cells, fungal cells from the three morphotypes were incubated with endoglycosidase H (New England BioLabs, Ipswich, MA, USA) or subjected to β-elimination to remove cell wall *N*-linked or *O*-linked glycans, respectively, using previously reported methodologies [37]. For the artefactual exposure of the inner cell wall layer at the cell surface, cells were heat-killed (HK). For this, fungal cells were incubated at 60 °C for 2 h, and the absence of fungal growth was demonstrated by incubating HK cells in YPD plates, pH 4.5, at 28 °C for 5 days [20].

### 2.2. Ethics Statement

The use of human cells in this research was approved by Universidad de Guanajuato through its Ethics Committee. The approval reference given to this study is CEPIUG-P22-2022. Venous blood samples were withdrawn from healthy adult volunteers after information about the study was disclosed and written informed consent was signed. This study was conducted following the Declaration of Helsinki.

### 2.3. Isolation of Human Granulocytes

Venous blood samples were mixed with Histopaque-1077 (Sigma-Aldrich, Saint Louis, MO, USA), and cells were separated via differential centrifugation as reported elsewhere [38]. The granulocytes/red blood cell phase at the bottom of the gradient were collected and suspended in 50 mL of lysis reagent (154.4 mM ammonium chloride, 10 mM potassium bicarbonate, and 97.3 mM EDTA tetrasodium salt) [39]. Then, cells were suspended in RPMI-1640 Dutch modification (Sigma-Aldrich), and the concentration was adjusted at 5 × 10^6^ cells mL^−1^. Cells were inspected under bright light microscopy to assess degranulation, which was absent in all preparations. Under these conditions, 96.0 ± 0.3%, 3.0 ± 0.1%, and 1.0 ± 0.2% cells were neutrophils, eosinophils, and basophils, respectively.

### 2.4. Cytokine Stimulation

Interactions were performed in U-bottom 96-well microplates, in a total volume of 200 µL. Each well contained 5.0 × 10^5^ granulocytes and 1.0 × 10^5^ fungal cells. The plates were incubated for 24 h at 37 °C with 5% (*v*/*v*) CO_2_ and centrifuged for 10 min at 1800× *g* at 4 °C, and supernatants were saved and kept at −20 °C until used. Secreted cytokines were quantified via ELISA using the Standard ABTS ELISA Development kits (Peprotech, Cranbury, NJ, USA) for human tumor necrosis factor-alpha (TNFα), interleukin 6 (IL-6), interleukin 8 (IL-8), and interleukin 10 (IL-10). Mock wells, where only human cells were included, were used as controls in all cytokine quantifications. The readings obtained from these control wells were deducted from all the experimental wells.

In some experiments, human cells were preincubated for 60 min at 37 °C with any of the following compounds: 200 μg mL^−1^ laminarin (Sigma-Aldrich) [40]; 10 μg mL^−1^ anti-TLR4 antibody (Santa Cruz Biotechnology, Dallas, TX, USA sc-293072); 10 μg mL^−1^ anti-TLR2 antibody (Thermo-Fisher Scientific, Waltham, MA, USA 16-9922-82); 10 μg mL^−1^ anti-CD11b antibody (CR3, Thermo Fisher Scientific, MA5-16528); isotype-matched, irrelevant IgG1 antibody (10 μg mL^−1^, Santa Cruz Biotechnology, Cat. No. sc-52003, used as a control in experiments where TLR4 was blocked), 10 μg mL^−1^ IgG2aκ antibody (Thermo-Fisher Scientific, 14-4724-85, to control experiments where TLR2 was blocked); and 10 μg mL^−1^ IgG2 antibody (R&D, Minneapolis, MN, USA, Cat. No. MAB9794, to control experiments where CD11b was blocked) [20,21]. Despite the system being LPS-free, all the interactions were performed in the presence of 5 μg mL^−1^ polymyxin B (Sigma-Aldrich) [41,42].

### 2.5. Cytokine Stimulation

For fungal labeling, cells were incubated with 1 mg mL^−1^ Acridine Orange (Sigma-Aldrich) for 30 min at room temperature, the excess dye was washed with PBS, and the cell concentration was adjusted at 3 × 10^7^ cells mL^−1^ [43]. Six-well plates were used to perform the interactions at an immune cell: fungus ratio of 1:6 in 800 µL DMEM medium (Sigma-Aldrich). The plates were incubated for 2 h at 37 °C and 5% (*v*/*v*) CO_2_ [37], and immune cells were detached from plates with chilled PBS and incubated with 1.25 mg mL^−1^ Trypan Blue [44]. The phagocytic event was analyzed via cytometry using a FACSCanto II system (Becton Dickinson, Franklin Lakes, NJ, USA). Fifty thousand events were collected per sample through the FL1 and FL2 channels, which were previously calibrated with non-labeled immune cells [37,43,44]. Laminarin and the antibodies listed in Section 2.4 were used in preincubation experiments as described.

### 2.6. Analysis of Neutrophil Extracellular Traps

The analysis of NETs was performed as previously described [45], measuring the nucleic acids released into the extracellular compartment. Human granulocytes were suspended at a final concentration of 4 × 10^7^ cells mL^−1^ in RPMI 1640, 175 µL was placed in the 96-well plates previously coated with 1% bovine serum albumin, and cells were incubated for 30 min at 37 °C and 5% CO_2_. Next, 25 µL of fungal cells adjusted at 4 × 10^8^ cells mL^−1^ was added to the wells, and interactions were incubated for 4 h at 37 °C and 5% CO_2_. Then, the plates were centrifuged, and the supernatant was collected and used to quantify nucleic acids via spectrophotometry at 260 nm in a NanoDrop One (Thermo Fisher Scientific). As a negative control, human cells were incubated only with PBS, while as a positive control, neutrophils were incubated with yeast-like cells from *C. albicans* SC5314. Alternatively, after the cell–cell interactions, the supernatants were collected, and fungal cells were stained with 20 µg mL^−1^ calcofluor white (Sigma-Aldrich) for 30 min at room temperature. Then, cells were washed with PBS and laid down in Poly-L-lysine-coated slides, cells were fixed with 4% formaldehyde, and then cells were stained with 10 µg mL^−1^ ethidium bromide. Cells were inspected under fluorescent microscopy, using a Zeiss Axioscope-40 microscope equipped with an Axiocam MRc camera (Zeiss, Oberkochen, Germany).

### 2.7. Statistical Analysis

Analyses were performed in GraphPad Prism 6 software, using the Mann–Whitney U and Kruskal–Wallis tests, with a significance level set at *p* < 0.05. All experiments were carried out with samples from eight healthy donors assayed in duplicate. The results are shown as means and standard deviations.

## 3. Results

### 3.1. Differential Cytokine Production by Human Granulocytes Stimulated with Conidia, Germlings, and Yeast-like Cells from Sporothrix schenckii, Sporothrix brasiliensis, and Sporothrix globosa

**Figure 1:**
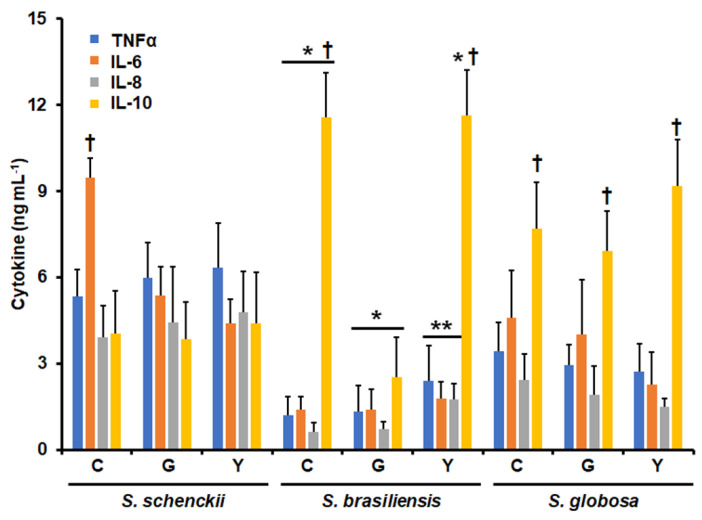
Cytokine production by human granulocytes co-incubated with conidia, germlings, or yeast-like cells from *Sporothrix schenckii*, *Sporothrix brasiliensis*, and *Sporothrix globosa*. Human granulocytes and fungal cells were co-incubated for 24 h; the supernatants were saved and used to determine the levels of secreted cytokines via ELISA. * *p* < 0.05 when compared with cytokines stimulated by *S. schenckii* or *S. globosa*. ** *p* < 0.05 when compared with cytokines stimulated by *S. schenckii*. † *p* < 0.05 when compared with the cytokine levels of the same morphology and the same species. C, conidia; G, germlings; Y, yeast-like cells. Results are shown as mean ± standard deviation from data generated with samples from eight donors analyzed in duplicate.

Human granulocytes were co-incubated with cells from the three species under analysis, and secreted TNFα, IL-6, IL-8, and IL-10 were quantified using ELISA. We selected these cytokines because the main component of the granulocyte population was neutrophils, and these cytokines have been previously demonstrated to be highly produced by these immune cells during sepsis and interaction with different pathogens [26,46]. Figure 1 shows the results of the cytokine quantification, and it is easy to see species-specific cytokine profiles. For conidia, the three species stimulated different levels of the four cytokines, with *S. schenckii* cells being associated with the highest levels of TNFα, IL-6, and IL-8, followed by *S. globosa* and *S. brasiliensis* (Figure 1). Contrary to this observation, the highest IL-10 levels were associated with *S. brasiliensis* conidia, followed by *S. globosa* and *S. schenckii* (Figure 1). For germlings, the three proinflammatory cytokines followed the same trend observed in conidia, but IL-10 stimulation was different, with the highest levels being found in the cells stimulated with *S. globosa*, followed by *S. schenckii* and *S. brasiliensis* (Figure 1). For yeast-like cells, once again, the highest levels of TNFα, IL-6, and IL-8 were associated with the cells stimulated with *S. schenckii*, while similar levels of the three cytokines were stimulated by both *S. globosa* and *S. brasiliensis* (Figure 1). The highest IL-10 levels were stimulated by *S. brasiliensis* yeast-like cells, followed by *S. globosa* and *S. schenckii* cells (Figure 1). There were also differences in the levels of cytokines stimulated when compared for each morphology and species. *S. schenckii* germlings and yeast-like cells stimulated similar levels of the four cytokines, but IL-6 was significantly higher than the other cytokines when conidia were used in the stimulations (Figure 1). For both *S. brasiliensis* conidia and yeast-like cells, IL-10 was significantly higher than the other cytokines, whilst no significant differences were observed in the cells stimulated with germlings. Finally, for the three *S. globosa* morphologies, the level of IL-10 was higher than that of the other three cytokines (Figure 1). Collectively, these data indicate that the interaction of granulocytes with *Sporothrix* cells is morphology- and species-specific.

**Figure 2:**
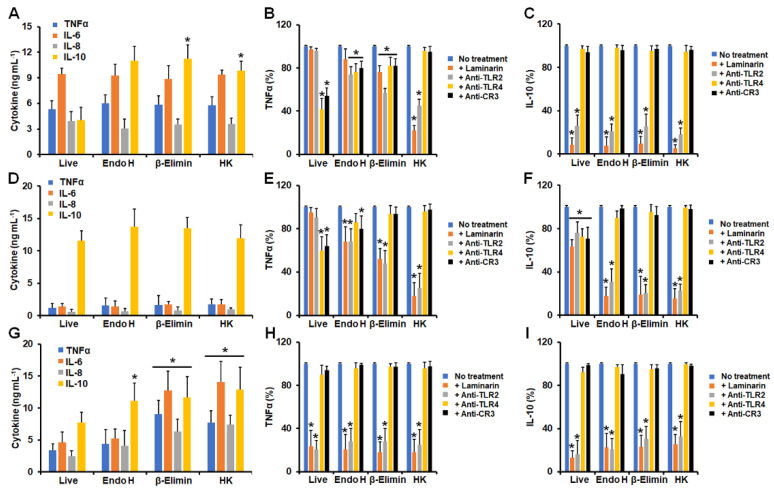
Cytokine stimulation by *Sporothrix schenckii*, *Sporothrix brasiliensis*, and *Sporothrix globosa* conidia interacting with human granulocytes. In (**A**,**D**,**G**), human cells were co-incubated with conidia for 24 h at 37 °C and 5% (*v*/*v*) CO_2_; supernatants were saved and used for TNFα, IL-6, IL-8, and IL-10 quantification. In (**B**,**C**,**E**,**F**,**H**,**I**), human cells were preincubated with 200 μg mL^−1^ laminarin or 10 μg mL^−1^ of any of the following antibodies: anti-TLR2, anti-TLR4, or anti-complement receptor 3 (CR3), before co-incubation with conidia. No treatment, cells preincubated with PBS. In all cases, 100% corresponds to the system with no treatment, and the absolute values are like those shown in panels (**A**,**D**,**G**). Endo-H, conidia treated with endoglycosidase H; β-Elimin, conidia subjected to β-elimination; HK, fungal cells inactivated by heat. Panels (**A**–**C**) correspond to *Sporothrix schenckii* conidia; (**D**–**F**) correspond to *Sporothrix brasiliensis* conidia; and (**G**–**I**) correspond to *Sporothrix globosa* conidia. In (**A**,**D**,**G**), * *p* < 0.05 when compared to cytokine levels stimulated by live cells. In (**B**,**C**,**E**,**F**,**H**,**I**), * *p* < 0.05 when compared to the no-treatment condition of the same strain. Results are shown as mean ± standard deviation from data generated with samples from eight donors analyzed in duplicate.

Next, we assessed the contribution of some cell wall components and pattern recognition receptors (PRRs) to the cytokine stimulation of the different *Sporothrix* morphologies. In all cases, we removed *N*-linked glycans via treatment with endoglycosidase H (Endo H) [37,41], and we removed *O*-linked glycans via β-elimination [20,47] or inactivation with heat, as this treatment artifactually exposes inner cell wall components at the cell surface, such as glucans and chitin [20,48,49]. Under these treatments, *S. schenckii* conidia stimulated similar levels of TNFα, IL-6, and IL-8 in live cells, but IL-10 levels were increased upon β-elimination or in heat-killed (HK) cells (Figure 2A). In contrast, these treatments did not affect the cytokine profile stimulated by *S. brasiliensis* conidia under the cell-wall-perturbing treatments (Figure 2D). In the case of *S. globosa* conidia, the endo-H treatment positively affected IL-10 production, whilst β-elimination and HK cells stimulated higher levels of the four cytokines analyzed (Figure 2G).

Next, we took the levels of TNFα and IL-10, as signature cytokines of proinflammatory and anti-inflammatory responses, and used them to monitor the contribution of some PRRs to the stimulation of these cytokines. We blocked dectin-1 with the specific antagonist laminarin [22,23,50], whereas TLR2, TLR4, and complement receptor 3 (CR3), some of the main receptors found on the granulocyte surface [26,51], were blocked with specific monoclonal antibodies. TNFα stimulation by *S. schenckii* conidia was significantly dependent on TLR4 and CR3, and this dependency was partially lost in endoH and β-eliminated cells and lost in HK cells (Figure 2B). As compensation, cytokine production was in addition dependent on TLR2 in the case of endo H cells, and it was dependent on dectin-1 and TLR2 in β-eliminated and HK cells (Figure 2B). IL-10 production was significantly dependent on dectin-1 and TLR2, regardless of the treatment applied to *S. schenckii* conidia (Figure 2C). For *S. brasiliensis* conidia, TNFα stimulation occurred via TLR4 and CR3, but this changed when cells were treated with Endo H, β-eliminated, or treated with heat, with cytokine production occurring through dectin-1 and TLR2 in these three cases (Figure 2E). IL-10 production occurred on the four analyzed receptors when live conidia were used in the experiments, but upon endo-H, β-elimination, or inactivation by heat, IL-10 production occurred via dectin-1 and TLR2 (Figure 2F). In the case of *S. globosa* conidia, both TNFα and IL-10 production was dependent on dectin-1 and TLR2, regardless of the conidia treatment (Figure 2H,I). Control experiments, where human cells were preincubated with irrelevant antibodies, showed similar cytokine values to non-preincubated cells.

**Figure 3:**
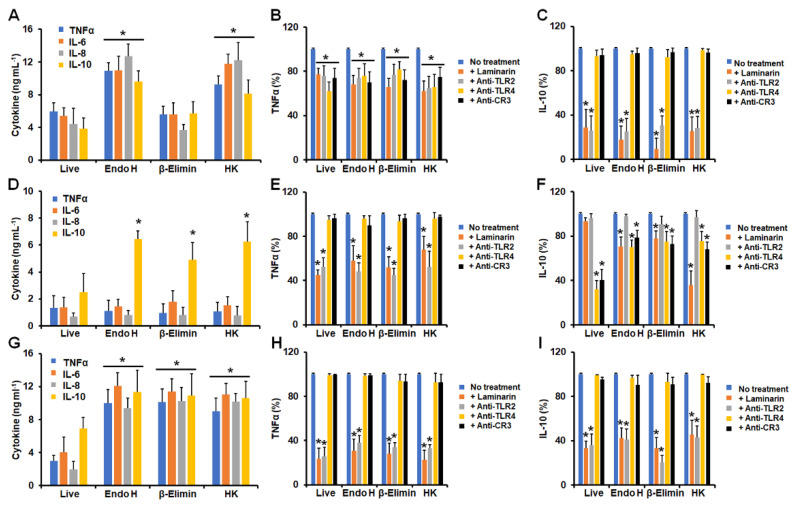
Cytokine stimulation by *Sporothrix schenckii*, *Sporothrix brasiliensis*, and *Sporothrix globosa* germlings interacting with human granulocytes. In (**A**,**D**,**G**), human cells were co-incubated with germlings for 24 h at 37 °C and 5% (*v*/*v*) CO_2_; supernatants were saved and used for TNFα, IL-6, IL-8, and IL-10 quantification. In (**B**,**C**,**E**,**F**,**H**,**I**), human cells were preincubated with 200 μg mL^−1^ laminarin or 10 μg mL^−1^ of any of the following antibodies: anti-TLR2, anti-TLR4, or anti-complement receptor 3 (CR3), before co-incubation with conidia. No treatment, cells preincubated with PBS. In all cases, 100% corresponds to the system with no treatment, and the absolute values are like those shown in panels (**A**,**D**,**G**). Endo-H, germlings treated with endoglycosidase H; β-Elimin, germlings subjected to β-elimination; HK, fungal cells inactivated by heat. Panels (**A**–**C**) correspond to *Sporothrix schenckii* germlings; (**D**–**F**) correspond to *Sporothrix brasiliensis* germlings; and (**G**–**I**) correspond to *Sporothrix globosa* germlings. In (**A**,**D**,**G**), * *p* < 0.05 when compared to cytokine levels stimulated by live cells. In (**B**,**C**,**E**,**F**,**H**,**I**), * *p* < 0.05 when compared to the no-treatment condition of the same strain. Results are shown as mean ± standard deviation from data generated with samples from eight donors analyzed in duplicate.

In the case of germlings, *N*-linked glycan trimming positively affected the cytokine production stimulated by *S. schenckii* and *S. globosa* cells, and in *S. brasiliensis* germlings, only IL-10 production was positively affected after treatment with endo H (Figure 3A,D,G). A similar trend was observed when these cells were β-eliminated or HK (Figure 2D). The removal of *O*-linked glycans from the *S. schenckii* germling did not affect cytokine production, but for *S.globosa*, the four cytokines significantly increased (Figure 3A,G). In both *S. schenckii* and *S. globosa* germlings, cytokine levels increased when HK cells were used for stimulation (Figure 3A,G). For the three species, TNFα stimulation was dependent on dectin-1 and TLR2, but in *S. schenckii,* it was also dependent on TLR4 and CR3 (Figure 3B,E,H). IL-10 was stimulated via dectin-1 and TLR2 for both *S. schenckii* and *S. globosa* germlings, but in *S. brasiliensis,* it was stimulated via TLR4 and CR3 (Figure 3C,F,I). In addition, endo-H, β-eliminated, and HK *S. brasiliensis* germlings stimulated IL-10 through dectin-1 (Figure 3F). Control experiments, where human cells were preincubated with irrelevant antibodies, showed similar cytokine values to non-preincubated cells.

**Figure 4:**
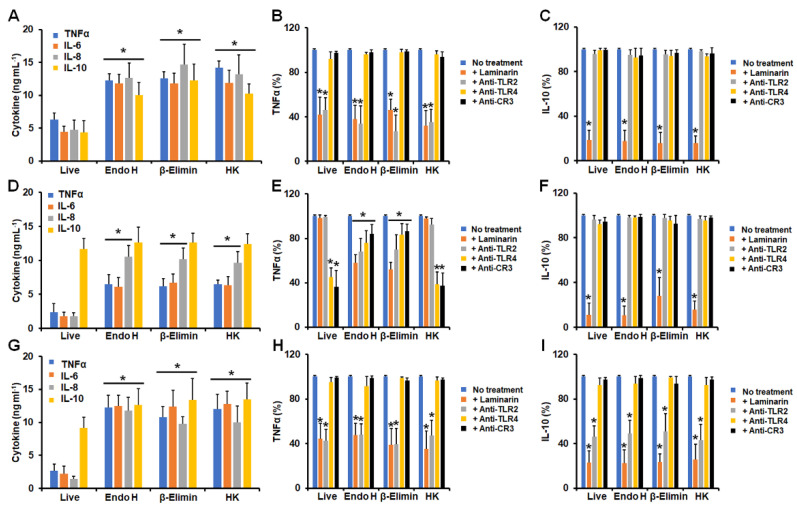
Cytokine stimulation by *Sporothrix schenckii*, *Sporothrix brasiliensis*, and *Sporothrix globosa* yeast-like cells interacting with human granulocytes. In (**A**,**D**,**G**), human cells were co-incubated with yeast-like cells for 24 h at 37 °C and 5% (*v*/*v*) CO_2_; supernatants were saved and used for TNFα, IL-6, IL-8, and IL-10 quantification. In (**B**,**C**,**E**,**F**,**H**,**I**), human cells were preincubated with 200 μg mL^−1^ laminarin or 10 μg mL^−1^ of any of the following antibodies: anti-TLR2, anti-TLR4, or anti-complement receptor 3 (CR3), before co-incubation with yeast-like cells. No treatment, cells preincubated with PBS. In all cases, 100% corresponds to the system with no treatment, and the absolute values are like those shown in panels (**A**,**D**,**G**). Endo-H, yeast-like cells treated with endoglycosidase H; β-Elimin, yeast-like cells subjected to β-elimination; HK, fungal cells inactivated by heat. Panels (**A**–**C**) correspond to *Sporothrix schenckii* yeast-like cells; (**D**–**F**) correspond to *Sporothrix brasiliensis* yeast-like cells; and (**G**–**I**) correspond to *Sporothrix globosa* yeast-like cells. In (**A**,**D**,**G**), * *p* < 0.05 when compared to cytokine levels stimulated by live cells. In (**B**,**C**,**E**,**F**,**H**,**I**), * *p* < 0.05 when compared to the no-treatment condition of the same strain. Results are shown as mean ± standard deviation from data generated with samples from eight donors analyzed in duplicate.

When yeast-like cells were used in this kind of interaction, the modification of the cell wall by endo H treatment, β-elimination, or heat treatment positively affected the stimulation of TNFα, IL-6, IL-8, and IL-10 in the cases of *S. schenckii* and *S. globosa* (Figure 4A,G). *S. brasiliensis* yeast-like cells followed a similar trend, but in this case, IL-10 stimulation was not related to any of the treatments applied to fungal cells (Figure 4D). When the contribution of PRRs was analyzed, we found that TNFα and IL-10 stimulation by *S. schenckii* yeast-like cells was dectin-1-dependent (Figure 4B,C), but the former was also dependent on TLR2 (Figure 4B). Live and HK *S. brasiliensis* yeast-like cells stimulated TNFα via TLR4 and CR3, but this dependency was partially lost in endo H and β-eliminated cells, and there was additional involvement of dectin-1 and TLR2 (Figure 4E). IL-10 was dependent solely on dectin-1 though (Figure 4F). Finally, both TNFα and IL-10 production stimulated by *S. globosa* yeast-like cells was dectin-1- and TlR2-dependent (Figure 4I). Control experiments, where human cells were preincubated with irrelevant antibodies, showed similar cytokine values to non-preincubated cells.

### 3.2. Differential Phagocytosis of Conidia and Yeast-like Cells from Sporothrix schenckii, Sporothrix brasiliensis, and Sporothrix globosa

**Figure 5:**
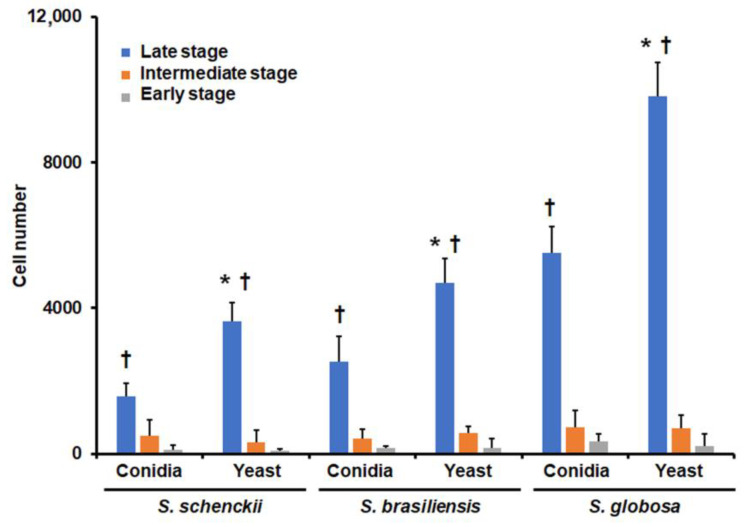
Phagocytosis of *Sporothrix schenckii*, *Sporothrix brasiliensis*, and *Sporothrix globosa* conidia and yeast-like cells by human granulocytes. Fungal and human cells interacted for 2 h at 37 °C and 5% (*v*/*v*) CO_2_ before human cells were analyzed using flow cytometry. Cells were selected for quantification when interacting with at least one fungal cell. * *p* < 0.05 when compared to conidia from the same species. † *p* < 0.05 when compared with the same morphology of the other two fungal species. Data are shown as means ± SD from eight donors analyzed in duplicate.

Next, we analyzed the ability of human granulocytes to phagocyte these fungal cells. We omitted the analysis of germlings because of the technical limitations of our strategy to analyze uptake via cytometry, as this cell morphology is capable of clotting the internal piping of a flow cytometer [52]. The strategy used here has been previously validated for the analysis of the uptake of conidia and blastoconidia, and, depending on the fluorescence associated with cells, these can be classified as in the early, intermediate, or late stage of phagocytosis [37,43]. Here, no significant differences were observed in human cells in the early and intermediate stages of the phagocytosis of the conidia and yeast-like cells of the three fungal species under analysis (Figure 5). However, significant differences were observed in the late stage (Figure 5). *S. schenckii* conidia and yeast-like cells were the lesser phagocytosed cells, followed by both morphologies of *S. brasiliensis* and finally *S. globosa* cells, which were the most phagocytosed (Figure 5). In addition, the three species followed the same uptake trend, where yeast-like cells were more readily phagocytosed than conidia (Figure 5).

**Figure 6:**
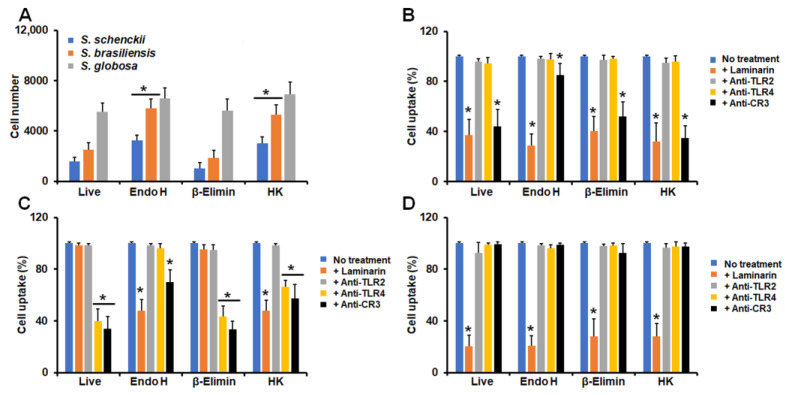
Contribution of cell wall components and pattern recognition receptors to the phagocytosis of *Sporothrix schenckii*, *Sporothrix brasiliensis*, and *Sporothrix globosa* conidia by human granulocytes. In (**A**), human granulocytes were incubated for 2 h at 37 °C, and phagocytosis was analyzed using flow cytometry. In (**B**–**D**), human granulocytes were preincubated with 200 μg mL^−1^ laminarin or 10 μg mL^−1^ of any of the following antibodies: anti-CR3, anti-TLR2, or anti-TLR4. Then, phagocytosis was analyzed as described in the Materials and Methods Section. All the interactions were performed in the presence of 5 μg mL^−1^ polymyxin B. No treatment, cells preincubated with PBS. Results correspond to cells in the late stage of phagocytosis. For all cases, 100% corresponds to human cells preincubated with PBS, and the absolute values are similar to those shown in panel (**A**). Endo H, conidia treated with endoglycosidase H; β-Elimin, conidia treated by β-elimination; HK, heat-killed conidia. In (**A**), * *p* < 0.05 when compared to live cells. In (**B**–**D**), * *p* < 0.05 when compared to the no-treatment condition of the same strain. In B, experiments were performed with *S. schenckii* conidia. In (**C**), experiments were performed with *S. brasiliensis* conidia, while in (**C**), *S. globosa* conidia were used. In all panels, data are shown as means ± SD from eight donors analyzed in duplicate.

Similar to our analysis of cytokine production, we also determined the contribution of some cell wall components and PRRs to the phagocytic process. Since the majority of granulocytes were in the late stage of phagocytosis in our experimental setting, we only analyzed cells at this stage. In the case of conidia, endo-H-treated cells and HK cells from *S. schenckii* and *S. brasiliensis* were more phagocytosed than live cells, but not when conidia were β-eliminated (Figure 6A). *S. globosa* conidia were similarly phagocytosed, regardless of the treatment applied to cells (Figure 6A). In all cell treatments, *S. schenckii* conidia phagocytosis was dependent on both dectin-1 and CR3, but in endo-H-treated cells, this dependency was diminished when compared to live and other treated cells (Figure 6B). Live, β-eliminated, and HK *S. brasiliensis* conidia were phagocytosed via TLR4 and CR3, but the uptake of endo-H-treated cells occurred via dectin-1 and CR3 (Figure 6C). Finally, *S. globosa* conidia was phagocytosed via dectin-1, regardless of the treatment applied to cells (Figure 6D). Granulocytes preincubated with irrelevant antibodies showed a similar uptake ability to non-preincubated cells.

**Figure 7:**
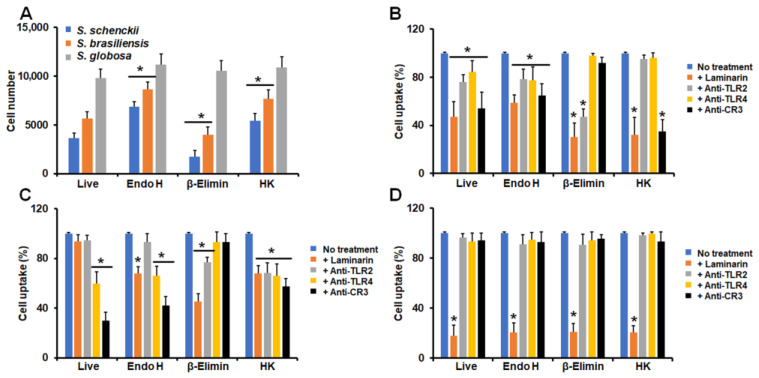
Contribution of cell wall components and pattern recognition receptors to the phagocytosis of *Sporothrix schenckii*, *Sporothrix brasiliensis*, and *Sporothrix globosa* yeast-like cells by human granulocytes. In (**A**), human granulocytes were preincubated with yeast-like cells and incubated for 2 h at 37 °C, and phagocytosis was analyzed using flow cytometry. In (**B**–**D**), human granulocytes were preincubated with 200 μg mL^−1^ laminarin or 10 μg mL^−1^ of any of the following antibodies: anti-CR3, anti-TLR2, or anti-TLR4. Then, phagocytosis was analyzed as described in the Materials and Methods Section. All the interactions were performed in the presence of 5 μg mL^−1^ polymyxin B. No treatment, cells preincubated with PBS. Results correspond to cells in the late stage of phagocytosis. For all cases, 100% corresponds to human cells preincubated with PBS, and the absolute values are similar to those shown in panel (**A**). Endo H, yeast-like cells treated with endoglycosidase H; β-Elimin, yeast-like cells treated by β-elimination; HK, heat-killed yeast-like cells. In (**A**), * *p* < 0.05 when compared to live cells. In (**B**–**D**), * *p* < 0.05 when compared to the no-treatment condition of the same strain. In (**B**), experiments were performed with *S. schenckii* yeast-like cells. In (**C**), experiments were performed with *S. brasiliensis* yeast-like cells, while in (**C**), *S. globosa* yeast-like cells were used. In all panels, data are shown as means ± SD from eight donors analyzed in duplicate.

When yeast-like cells were used in the interactions with granulocytes, we found that *S. schenckii* and *S. brasiliensis* cells were more phagocytosed when treated with endo H or with heat than live cells, but β-eliminated cells showed lower levels of uptake than the live control cells (Figure 7A). None of the treatments applied to fungal cells affected the ability of human granulocytes to phagocyte *S. globosa* yeast-like cells (Figure 7A). The uptake of *S. schenckii* yeast-like cells by human granulocytes was dependent on dectin-1, TLR2, TLR4, and CR3 in both live and endo-H treated cells (Figure 7A). However, in the case of β-eliminated cells, the uptake occurred via dectin-1 and TLR2, while HK cells were phagocytosed through dectin-1 and CR3 (Figure 7B).

Live *S. brasiliensis* yeast-like cells were phagocytosed via TLR4 and CR3, and these receptors, along with dectin-1, participated in the phagocytosis of endo-H-treated *S. brasiliensis* cells (Figure 7B). Both dectin-1 and TLR2 participated in the uptake of β-eliminated yeast-like cells, whereas the four receptors under analysis participated in the phagocytosis of these cells (Figure 7C). Similar to conidia, the *S. globosa* yeast-like cells were phagocytosed through a dectin-1-dependent mechanism, regardless of the cell treatment applied to fungal cells (Figure 7D). Granulocytes preincubated with irrelevant antibodies showed a similar uptake ability to non-preincubated cells.

### 3.3. Stimulation of Neutrophil Extracellular Traps by Sporothrix schenckii, Sporothrix brasiliensis, and Sporothrix globosa

**Figure 8:**
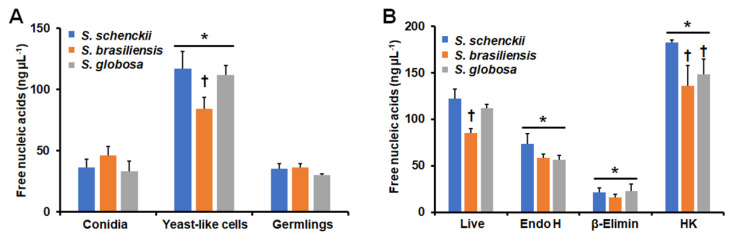
Stimulation of neutrophil extracellular traps by *Sporothrix schenckii*, *Sporothrix brasiliensis*, and *Sporothrix globosa*. In (**A**), human granulocytes and conidia, yeast-like cells or germlings of *S. schenckii*, *S. brasiliensis,* or *S. globosa* were placed in a MOI 1:10 and incubated for 4 h at 37 °C and 5% CO_2_. Then, plates were centrifuged, and supernatants were used to quantify nucleic acids by reading absorbance at 260 nm. In (**B**), similar experiments to those described in panel (**A**) were performed but only using yeast-like cells. Endo H, yeast-like cells treated with endoglycosidase H; β-Elimin, yeast-like cells treated by β-elimination; HK, heat-killed yeast-like cells. In (**A**), * *p* < 0.05 when compared to conidia or germlings; † *p* < 0.05 when compared with cells of the same morphology. In (**B**), * *p* < 0.05 when compared to live cells; † *p* < 0.05 when compared with cells of the same morphology. Results are shown as mean ± standard deviation from data generated with samples from eight donors analyzed in duplicate.

**Figure 9:**
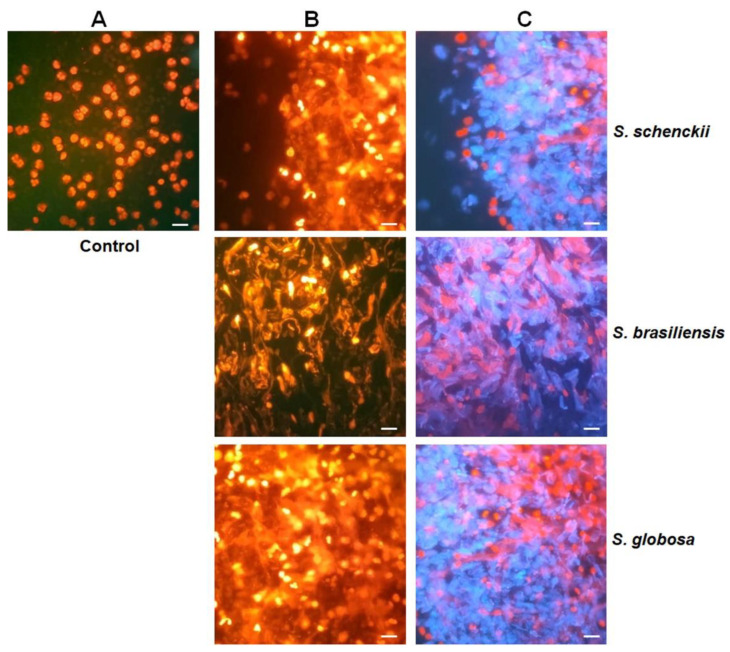
Representative images of neutrophil extracellular traps stimulated by *Sporothrix schenckii*, *Sporothrix brasiliensis*, and *Sporothrix globosa* yeast-like cells. Human granulocytes and yeast-like cells of *S. schenckii*, *S. brasiliensis,* or *S. globosa* were placed in a MOI 1:10 and incubated for 4 h at 37 °C and 5% CO_2_. DNA was stained with ethidium bromide (panels **A**,**B**), while fungal cells were labeled with calcofluor white (**C**). Panel (**A**) corresponds to non-stimulated human granulocytes that were used as controls. Panels (**B**,**C**) correspond to the ethidium bromide and ethidium bromide plus calcofluor with staining, respectively. Scale bars = 20 µm.

Since neutrophils are the most abundant cell population in our granulocyte preparations, we next analyzed the ability of the fungal cells to stimulate NETs. We indirectly measured the ability to stimulate these traps by quantifying the nucleic acids released into the extracellular compartment, as this area is the main component of NETs [45]. Both conidia and germlings from the three species showed a similar ability to stimulate NETs (Figure 8B). On the contrary, yeast-like cells showed an increased ability to stimulate NETs, but *S. schenckii* and *S. globosa* were better stimuli than *S. brasiliensis* yeast-like cells (Figure 8A). Control cells only incubated with PBS released 12.9 ± 1.4 ng µL^−1^ nucleic acids, while human granulocytes incubated with *C. albicans* cells released 45.8 ± 8.8 ng µL^−1^ nucleic acids. These data indicate that yeast-like cells from the three *Sporothrix* species are better stimulants than the positive control, *C. albicans*. Since this morphology showed a high ability to stimulate NETs, we focused only on these cells and assessed the contribution of cell wall components. Endo H and β-eliminated cells from the three species showed a lower ability to stimulate NETs than live cells, but the lack of *O*-linked glycans was markedly different from the cells lacking *N*-linked glycans (Figure 8B). On the contrary, HK cells from the three fungal species showed an increased ability to stimulate NETs, but *S. schenckii* cells were a better stimulus than the other two fungal species (Figure 8B). Representative images of the NETS stimulated with yeast-like cells are shown in Figure 9.

## 4. Discussion

The study of the interaction between *Sporothrix* and human granulocytes remains scarce, despite the relevant roles of these immune cells in the first line of defense. Even though neutrophils were the main type of human cells in our preparations, formally, we cannot directly link our results solely to this type of immune cell. Here, we observed that the three *S. schenckii* morphologies showed the highest ability to stimulate proinflammatory cytokines, whereas the lowest levels were associated with *S. brasiliensis*. To the best of our knowledge, this is the first report of cytokine profiles stimulated by *Sporothrix* when interacting with human granulocytes. Interestingly, this cytokine profile is similar to that previously observed when these fungal cells interacted with human PBMCs and human monocyte-derived macrophages [20,22,23]. This trend, however, was not observed in the case of IL-10, which was highly stimulated by *S. globosa* cells interacting with human PBMCs and monocyte-derived macrophages [20,22,23], but here, *S. brasiliensis* was the species that stimulated the highest levels of this cytokine. Thus, these data suggest a basic response core in these human immune cells that has type-specific response signatures. In support of this, our cytokine profile with the three fungal species is different from that reported with *Sporothrix* cells interacting with human dendritic cells, where *S. globosa* was the most potent stimulant of proinflammatory cytokines [23].

Both *N*-linked and *O*-linked glycans were dispensable for proinflammatory cytokine stimulation by *S. schenckii* conidia, and they seem to play a masking role of inner wall components, as cells lacking any of these compounds stimulated higher IL-10 levels with no effect on proinflammatory cytokines. However, the role of these wall components is not as passive as mentioned, because in live *S. schenckii* and *S. brasiliensis* conidia cells, the main PRRs receptors involved in TNFα stimulation were TLR4 and CR3, which recognize rhamnose-containing glycans [21]. Proinflammatory stimulation was maintained in the system lacking *N*-linked or *O*-linked glycans because of the shifting of PRRs to dectin-1 and TLR2. In *S. globosa* conidia, this masking-only role of glycans is possible to conceive, because the removal of the compounds positively affected cytokine production, which was dectin-1- and TLR2-dependent in all the tested conditions. Interestingly, a similar PRR dependency was recently reported for cytokine production in human monocyte-derived macrophages [23].

In the case of germlings, *S. globosa* with no *N*-linked or *O*-linked glycans on the surface were better stimulants for cytokines than the untreated cells, but in all cases, stimulation occurred in a pathway dependent on dectin-1 and TLR2, suggesting that β-1,3-glucan is the main cell wall pathogen-associated molecular pattern involved in cytokine stimulation. These data are in line with previous cell wall characterization data that indicate that *S. globosa* has more β-1,3-glucans exposed at the cell surface than *S. schenckii* or *S. brasiliensis* [22,53]. IL-10 stimulation by *S. brasiliensis* germlings increased in cells lacking *N*-linked or *O*-linked glycans, but dependency on receptors changed from TLR4 and CR3 in nontreated cells to these receptors as well as dectin-1 in treated cells, suggesting that, for this species, glycans contribute to the stimulation of this anti-inflammatory cytokine, a different observation when compared to *S. schenckii* and *S. globosa*.

Contrary to the other morphologies, when yeast-like cells stimulated cytokines, these cytokines increased when cells were HK, β-eliminated, or treated with endo H, indicating that cell wall perturbations positively affected the immune sensing of the three fungal species by human granulocytes. Moreover, these results reinforce the idea that the cell walls of these species have morphology- and species-specific organization and composition [20,22,53,54]. Dectin-1 and TLR2 receptors were involved in cytokine stimulation by *S. schenckii* and *S. globosa* cells, suggesting a key role of β-1,3-glucans in sensing by granulocytes. These receptors were also involved in TNFα stimulation by *S. brasiliensis* yeast-like cells, but this role was shared with TLR4 and CR3, which is in line with observations in human PBMCs, where CR3 plays a differential role in the sensing of *S. schenckii* and *S. brasiliensis* yeast-like cells [21]. Our results related to yeast-like cells contrast with those previously reported, where dectin-1 was found to be dispensable for the clearance of *S. schenckii* in an experimental model of sporotrichosis [55]; however, they are in line with recent observations that have placed dectin-1 as a central component of anti-*Sporothrix* innate immunity [20,22,23,56].

Regarding phagocytosis, here, yeast-like cells were more phagocytosed than conidia, an observation similar to that in previous studies dealing with *S. schenckii* [29]; however, *S. globosa* cells were more readily phagocytosed than the other species. Since β-1,3-glucan-dectin-1 interaction is one of the main players in fungal uptake by macrophages, including *C. albicans* and *Sporothrix* species [23,57], it is possible to suggest that this increased uptake of *S. globosa* cells may be related to the high β-1,3-glucan levels exposed at the cell surface [22,53]. This is further supported by the fact that the perturbation of conidia and yeast-like cell walls did not affect the fungal uptake, and the sole receptor found to be involved in phagocytosis was dectin-1. Similarly, dectin-1 was also involved in the phagocytosis of *S. schenckii* conidia and yeast-like cells, but like in the cytokine stimulation, this dependence was not observed for *S. brasiliensis*. Instead, TLR4 and CR3 were the main players for the phagocytosis of both conidia and yeast-like cells.

Thus far, NETs stimulated by *Sporothrix* cells are a subject scarcely studied, and there is only one report about NET stimulation by *S. globosa* cells [58]. Our result indicates that both conidia and yeast-like cells are capable of inducing NETs, with the latter being a better stimulant, even better than *C. albicans* cells, which is in line with the observation of *Sporothrix* extracellular components having a better ability to stimulate reactive oxygen species than *C. albicans* cells [30]. Interestingly, the loss of *O*-linked glycans significantly reduced the ability to stimulate NETs in the three fungal species, suggesting that this cell wall component is a major player in NET stimulation. Since, in endo-H-treated cells, NET stimulation was also reduced, but not at the levels related to β-eliminated cells, it is possible to suggest that both *N*-linked and *O*-linked glycans are relevant for NET stimulation, likely through a costimulatory pathway, as has been described in cytokine stimulation in other fungal pathogens [48,50,59,60,61]. Since NET formation increased in HK cells, the involvement of β-1,3-glucan via dectin-1 is also likely.

In conclusion, we report here that the morphologies of *S. schenckii*, *S. brasiliensis*, and *S. globosa* play a role during the interaction with human granulocytes, generating morphology- and species-specific cytokine profiles. Nevertheless, *S. brasiliensis* tended to stimulate an anti-inflammatory cytokine profile, whilst the other two species had a proinflammatory response. *S. globosa* cells were the most phagocytosed cells, which occurred through a dectin-1-dependent mechanism, while the uptake of *S. brasiliensis* mainly occurred via TLR4 and CR3. The *N*-linked and *O*-linked glycans and β-1,3-glucans are cell wall components that play a significant role in the interaction of these *Sporothrix* species with human granulocytes.

## Data Availability

Not applicable.
